# Observation of tumor-associated macrophages expression in gastric cancer and its clinical pathological relationship

**DOI:** 10.1097/MD.0000000000019839

**Published:** 2020-04-24

**Authors:** Qing Zhu, Xia Wu, Mingyang Tang, Ligao Wu

**Affiliations:** a Departments of Pathology, The First Affiliated Hospital of Bengbu Medical College; b Department of Pathology, Bengbu Medical College; c Class 2018, Clinical Pathology, The Graduate School, Bengbu Medical College; d Class 2016, School of Clinical Medicine, Bengbu Medical College, Anhui, China.

**Keywords:** CD16, CD163, gastric tumor, helicobacter pylori, tumor associated macrophage

## Abstract

The present study was designed to investigate the expression of tumor-associated macrophages (TAMs) in gastric cancer and its clinicopathological relationship. In addition, we also aimed to analyze the relationship between helicobacter pylori (HP) infection and TAMs in gastric cancer.

The protein expression of CD16 and CD163 in 90 gastric cancer tissues and 30 margin tissues was detected by immunohistochemistry. HP infection was detected in 90 gastric cancer tissues and 30 margin tissues by gram staining and immunohistochemistry.

There was no clear correlation between CD16 macrophages and gastric cancer. The density of CD163 macrophages was not correlated with the general condition of tumor patients, but with tumor size, tumor differentiation, lymphatic metastasis, depth of invasion and TNM stage. Additionally, the infection rate of HP in gastric cancer tissues was significantly higher.

In summary, TAMs are associated with tumor size, degree of differentiation, depth of invasion, lymph node metastasis and TNM stage, suggesting their critical role in the invasion and metastasis of gastric cancer.

## 1 Introduction

Gastric cancer is a relatively common malignant tumor, which is associated with a high incidence rate around the world, and a variety of factors may affect the pathogenesis of gastric cancer.^[1,2]^ In recent years, the tremendous changes in people's living standard, the gradually accelerated life pace, and the different diet structure from the past have promoted the occurrence of gastric cancer to a certain extent. According to research on the pathogenic factors of gastric cancer, Helicobacter pylori (HP) infection is the main cause of disease.^[3,4]^ According to the current statistics, gastric cancer has gradually displayed a younger tendency, with continuous developmental trend.^[3]^ The onset of this malignant tumor is relatively insidious, and it is easy to be mistaken for other diseases by patients, leading to missed diagnosis and unsatisfactory early diagnosis rate.^[5]^ As a malignant tumor, a variety of factors can affect the prognosis, including the degree of differentiation and therapeutic measures, which play an essential role in the patient quality of life and disease progression.^[6,7]^ The current diagnostic technology has been updated, the therapeutic approaches have been continuously improved, and standardized treatment has been gradually promoted in clinical practice.^[8,9]^ However, distant metastasis can also occur in early-stage gastric cancer, and postoperative recurrence is relatively more common, which leads to out-of-control disease and even threatens patient life.^[10,11]^ Therefore, great attention should be paid to this situation, and we must carefully analyze the mechanism of metastasis and recurrence of gastric cancer, so as to fundamentally solve this problem, find the optimal way to control diseases, prolong patient life, and improve their quality of life, which is of great clinical significance.^[12–14]^

In recent years, more research focuses on changes in the tumor microenvironment during the gastric cancer pathological processes, and scholars have reported the massive infiltration of macrophages, which is finally proved to be the tumor-associated macrophages (TAMs). Further studies investigate the various types of malignant tumors, including gastric cancer, and discovers the correlation with chronic inflammation,^[15–17]^ which was closely related to the prognosis for cancer patients. TAMs are related to the occurrence of tumors, which always plays a key role, and even participates in immune regulation.^[18–20]^ By the TAM labeling methods, previous studies suggest that, the differentiation degree is significantly and positively correlated with TAM density in ovarian cancer patients.^[21,22]^ In a study on bladder cancer,^[23]^ the general conditions of patients are collected and long-term follow-up is performed. In addition, the mRNA expression of CD163 is determined in the surgically resected malignant tumor tissues, which revealed that the mRNA expression of CD163 is significantly and negatively associated with survival rate.

Studies have shown that HP infection is obviously and positively correlated with the incidence of gastric cancer.^[24]^ Samples have been collected from patients in high-risk areas of gastric cancer, and the results show that the proportion of patients infected with HP is more than 50%.^[25,26]^ HP is closely associated with many gastric disorders, and this is possibly because that, the implantation of HP in the gastric mucosa promotes the conversion of nitrate, increases the number of carcinogens, and stimulates the mucosa of the stomach wall, thus leading to abnormal differentiation of gastric mucosa and the resultant occurrence of malignant tumors.^[25,26]^ HP is also the main cause of chronic gastritis. The persistent inflammation may induce the abnormal proliferation of mucosal cells under the combined action of other factors, subsequently causing the occurrence of malignant tumor.^[27]^ HP itself can also produce a number of toxic products, which can stimulate the carcinogenesis of gastric mucosa, and may be fully confirmed by the fact that the detection rate of these toxicants in patients with gastric cancer is significantly higher. Therefore, HP infection may be correlated with gastric cancer, and TAMs phenotype is also related to gastric cancer, thus, we are interested in whether there is a certain relationship between them.^[28]^ However, the impact of HP on phenotypic differentiation of TAMs remains unknown so far. In the present study, the gastric cancer and margin tissues were collected, and the protein expression of CD16 and CD163 was evaluated and compared. The HP infection in different tissues was detected to explore the effects of different factors on gastric cancer by combining patient survival. Our study sheds more lights on the mechanism, effective preventive measurements, the optimal therapeutic approaches to improve prognosis, which is of important clinical significance.

## 2 Materials and methods

### 2.1 Patients

Patients with confirmed diagnosis of gastric cancer were retrospectively selected from January 2011 to December 2012. All patients underwent surgical resection, and the surgically obtained gastric cancer tissues were routinely prepared into paraffin-embedded tissues (90 cases). In addition, the marginal tissues were taken as control group (30 cases). The general information of patients was collected, and no other anti-cancer treatments were performed before surgery.

The present study was conducted in accordance with the Declaration of Helsinki and was approved by the Ethics Committee of Bengbu Medical College. Written informed consent was obtained from all participants.

### 2.2 Immunohistochemistry (IHC) assay

The tissue samples obtained from patients were fixed in 10% neutral formalin and embedded with paraffin. Afterwards, the paraffin-embedded tissues was serially cut into 4-μm-thick slices, followed by Elivision staining. Meanwhile, blank control group was set using PBS and DAB for visualization. Primary antibodies against CD16 and CD163, and the IHC kit were purchased from Fuzhou Maixin Biotech Co., Ltd. (Fuzhou, China). The positive expression of CD16 and CD163 was observed in TAMs. CD16-postive macrophages were classified into high- and low-density groups according to the number in each high power field, where 6.4 was used as the cut-off value. Similarly, CD163-positive macrophages were also divided into high and low density groups according to the number in each high power field, where 16.5 was used as the cut-off value.

### 2.3 Gram staining

The samples were fixed in the 10% neutral formalin and embedded with paraffin. Afterwards, the paraffin-embedded tissues were serially cut into 4-μm-thick slices, followed by Gram staining. The slices were observed under microscope in different fields of view (10 to 20), and the number of HP-positive cells was counted for average. The average cut-off value was set at 20, where the value of over 20 indicated positive HP infection. HP was found in many gastric cancer tissues, and most of them were located in the mucosal part, and presented in the cell membrane and cytoplasm, with granular appearance and brownish yellow staining.

### 2.4 Quantitative real-time PCR

Total RNA was purified using the TRIzol reagent (Invitrogen) according to the manufacturer's instructions. Afterwards, the high quality RNA was used for first-strand cDNA synthesis through reverse transcription using the M-MLV reverse transcriptase (TaKaRa, China). Thereafter, quantitative real-time RT-PCR was performed in the Roche lightcycler (LightCycler 2.0) using the TaKaRa SYBR Green I kit. The thermal cycling conditions for PCR were as follows, at 95°C for 30 s, followed by 40 cycles of 95°C for 5 s, at 60°C for 20 s and at 72°C for 20 s, as well as the final extension at 40°C for 20 min. The following primers were used in this study: for CD16, RT primers: 5’-GTCGT-ATCCAGTGCAGGGTCCGAGGTATTCGCACTGG-ATACGACGACACTCACC-3’; forward: 5’-GCCGAAACAUUCAACGCUGUC-3’; reverse: 5’-CAGTGCA-GGGTCCGAGGT-3’. for CD163, RT primers: 5’-TGTCG-CGATCGAGCTAAATGTGAAACGCCGCGTCACC-TATGCTGCTGCGCTGAA-3’; forward: 5’-CTTCGGGCGAATGGCAGCACT-3’; reverse: 5’-ATGTGAC-AAATCCATCCG-3’. For U6, RT primers: 5’-GTCG-TATCCAGTGCAGGGTCCGAGGTATTCGCAC-TGGATACGACAAAATATGGAAC-3; forward: 5’-TGCGGGTGCTCCGCTTCGGCAGC-3’; reverse: 5-CAGTGCAGGGTCCGAGGT-3’. All primers were synthesized by Invitrogen (New York).

### 2.5 Statistical analysis

SPSS23.0 software was used for statistical analysis, and a difference of *P* < .05 indicated statistical significance. Kaplan-Meier and Log-rank test were employed for survival analysis. In addition, univariate and multivariate Cox regression model analyses were performed. The correlation between HP infection and polarization of TAMs was also tested through Spearman rank correlation test.

## 3 Results

### 3.1 Expression of TAMs in gastric cancer and margin tissues

**Figure 1:**
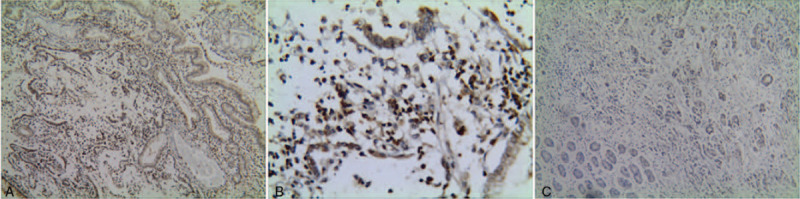
The expressions of CD16-positive macrophages in each group were detected by IHC assay. (A). High expression of CD16-positive macrophages in normal margin tissues (Elivision×100). (B). High expression of CD16-positive macrophages in early-stage gastric cancer (Elivision× 400). (C). Low expression of CD16-positive macrophages in advanced gastric cancer (Elivision× 400). IHC = immunohistochemistry.

**Figure 2:**
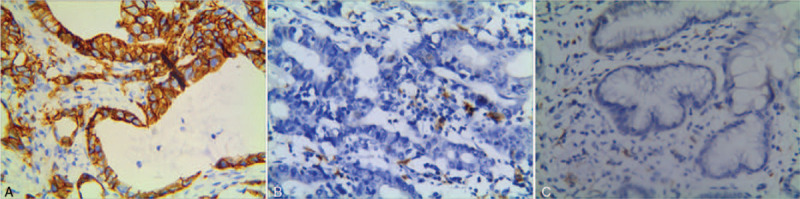
The expressions of CD16-positive macrophages in each group were detected by IHC assay. (A). Higher expression of CD163-positive macrophages in advanced gastric cancer (Elivision× 400). (B). Lower expression of CD163-positive macrophages in early-stage gastric cancer (Elivision× 400). (C). Low expression of CD163-positive macrophages in normal margin tissues (Elivision× 100).

CD16-positive macrophages were highly expressed in both tissues, and there was no significant difference between the 2 groups. However, the expression of CD163-positive macrophages was significantly up-regulated in gastric cancer tissues than that in margin tissues (*P* < .05) (Figs. 1 and 2).

### 3.2 The correlation between TAMs and clinical parameters

TAMs are distributed in gastric cancer tissues, however, the density varies in different standards, possibly due to the effects of various factors. The general information and pathological results from patients were examined, which showed that CD163-positive macrophage density was correlated with certain types of cells, as shown in Table 1. The cells showed high expression in gastric cancer tissues, however, with different densities. A variety of related factors were introduced, which revealed no definite correlation with the general condition and lesion site of patients (*P* > .05), but it had certain association with other factors. To be specific, tumor size was positively correlated with its density. The higher degree of tumor differentiation led to the higher density, and the higher density resulted in more lymphatic metastasis. Besides, the density increased with the increase in infiltration depth, and it was also significantly correlated with TNM staging. The density of CD16-positive macrophages was not significantly correlated with any of the related factors (*P* > .05).

**Table 1:**
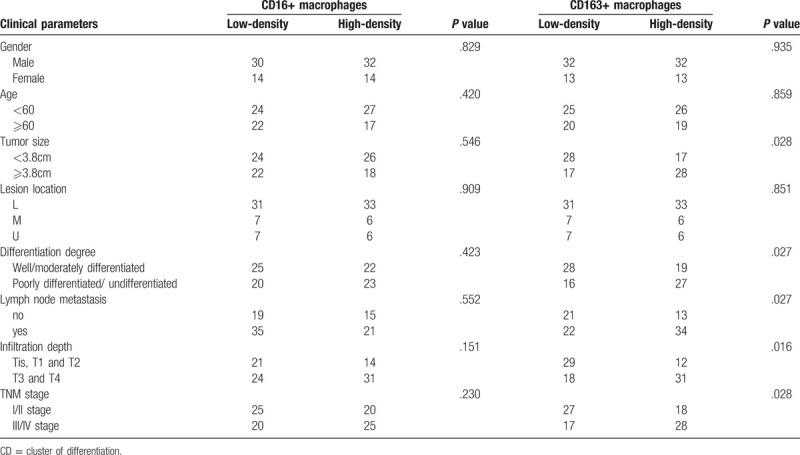
Relationship tumor-associated macrophage polarization and clinicopathological factors.

### 3.3 Prognostic factors in patients with gastric cancer

**Figure 3:**
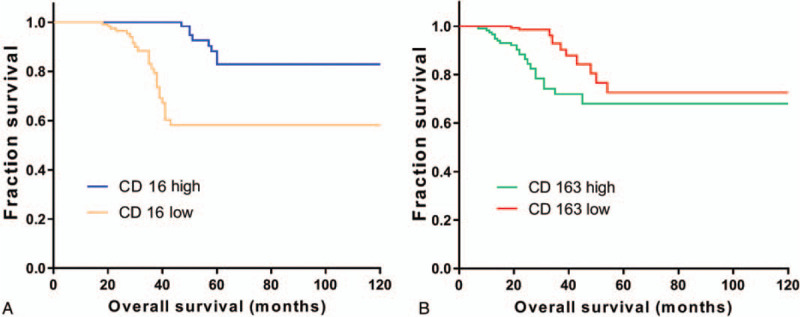
The overall survival in each group. The survival curves were plotted in patients with gastric cancer tumor associated with different expression of CD 16 (A) and CD 163(B) macrophage densities.

The subtypes of TAMs in gastric cancer tissues were definitely correlated with survival rate, as shown in Figure 3. According to CD16-positive macrophages, the density was obviously and positively correlated with survival time, with statistical significance between the two groups (*P* < .05). The density of CD163-positive macrophages was obviously and negatively correlated with survival time, with significant difference between the 2 groups (*P* < .05).

Cox regression analysis was used to determine the prognostic factors of gastric cancer, including tumor size, differentiation, metastasis, depth of infiltration, TNM stage, CD16- and CD163-positive macrophage density (*P* < .05), as well as other non-prognostic factors (*P* > .05) (shown in Table 2)

**Table 2:**
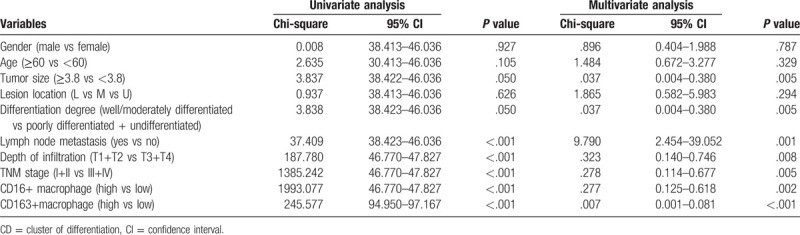
Cox regression analysis.

Cox regression analysis showed that tumor size, differentiation degree, lymph node metastasis, depth of infiltration, TNM stage, CD16- and CD163-positive macrophage density were the independent prognostic factors (*P* < .05) (Table 2). However, gender, age and location of gastric cancer were not associated with prognosis (*P* > .05).

### 3.4 HP infection in gastric cancer tissues

**Figure 4:**
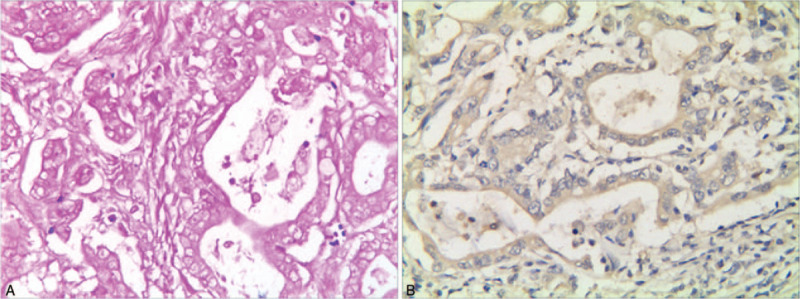
HP infection was detected in gastric cancer tissues in each group. (A) The Gram stain showed that HP was mainly in circular, giant body, short rod shap (×1000). (B). HP positive expression in gastric cancer was detected by IHC staining (Elivision× 400).

Gastric cancer samples were obtained, prepared for Gram staining and observed under microscope to confirm HP infection. There were 72 HP infection cases, with the positive rate of 72%, while the positive rate was 78% in IHC, and the pooled positive rate of these 2 methods was 70%. The HP infection rate was significantly higher in the gastric cancer group compared with that in control group (*P* < .05) (Fig. 4 and Table 3).

**Table 3:**
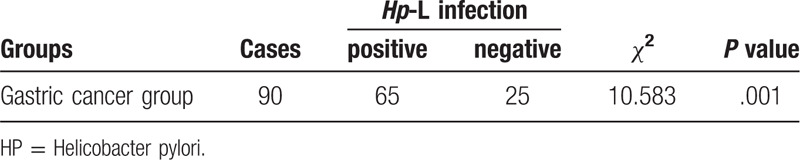
HP infection cases in different organizations.

### 3.5 Correlations of CD16 and CD163 protein expression with HP infection in gastric cancer tissues

**Figure 5:**
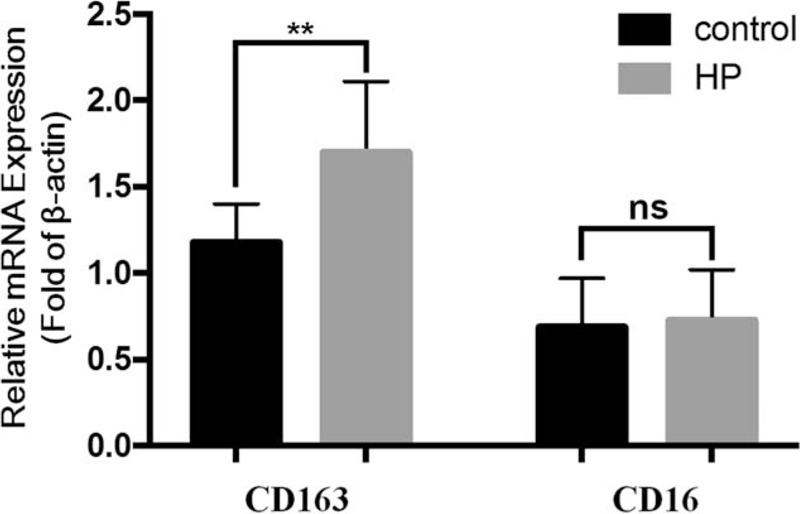
RT-PCR was used to detect the correlation between CD16 and CD163 gene expression and HP infection in gastric cancer tissues. The expression of CD16 and CD163 genes in HP-positive and HP-negative gastric cancer tissues was detected. Values were expressed as mean ± SD. ^∗∗^*P* < .01 compared with control group.

According to the presence or absence of HP infection, gastric cancer tissues were divided into HP-positive or HP-negative group. The positive expression rates of CD16 protein in HP-positive and HP-negative gastric cancer tissues were 50.77% and 48.00%, respectively. There was no significant correlation between CD16 protein expression and HP infection (*r* = 0.055, *P* = .814). In addition, the positive expression rates of CD163 protein in HP-positive and HP-negative gastric cancer tissues were 75.38% and 52.00%, separately, and the CD163 protein expression was positively correlated with HP infection (*r* = 4.556, *P* = .032) (Table 4 and Fig. 5).

**Table 4:**
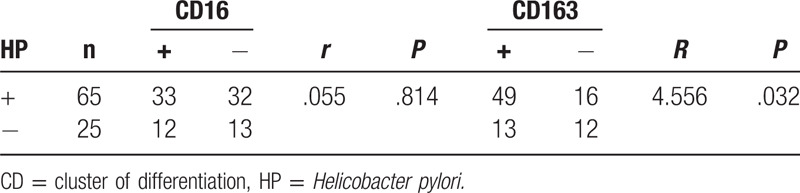
Expression of CD16 and CD163 proteins in HP-positive and HP-negative gastric cancer tissue.

## 4 Discussion

Recent studies show that there is massive infiltration of TAMs, which plays a role in the pathogenesis and progression of tumors.^[29]^ In the present study, the relationship between TAMs expression and pathogenic factors of gastric cancer was investigated. According to our results, there was no clear correlation between CD16 macrophages and gastric cancer. The density of CD163 macrophages was not correlated with the general condition of tumor patients, but with tumor size, tumor differentiation, lymphatic metastasis, depth of invasion and TNM stage. In addition, the infection rate of HP in gastric cancer tissues was significantly higher.

Macrophage is an important component of the innate immune response of human body, which can differentiate into different subtypes within different tissue microenvironments to exert various biological roles. Macrophages can be broadly divided into two categories, namely, M1-type classical activated macrophages and M2 type alternative activated ones. The marker CD16 is highly expressed in M1-type macrophages, whereas the marker CD163 shows high expression in M2-type macrophages. M1 macrophages in TAM can present antigens, kill microorganisms and tumor cells, whereas the M2 macrophages inhibit inflammatory responses, suppress immunity, promote angiogenesis and tumor cell growth, regulate the immune system.^[30,31]^ Macrophages can undergo phenotype change and functional polarization under different microenvironments, and thus exhibit different functions. Similar results are obtained in studies on gastric cancer.

Chronic inflammation is an important factor leading to tumor, and there is a correlation between these two. Long-term inflammatory stimuli can promote the abnormal proliferation of cells, leading to carcinogenesis and tumor progression, where the role of inflammation can not be ignored.^[32]^ Modern research has fully confirmed the close correlation between gastric cancer and chronic inflammation in gastric mucosa.^[33]^ Immune cells play an important role in the human body, which are a key part of the systemic protection. Typically, immune cells can produce antibodies during the anti-tumor process, thereby exerting the inhibitory effects and partially preventing tumor formation, as has been fully confirmed.^[34]^ However, the role of immune cells is bidirectional, which may exert a synergistic role in promoting the secretion of growth factors by tumor cells and in inhibiting the immune system to promote tumor formation. Multiple factors in this study are closely related to the prognosis for patients with gastric cancer.^[35]^ TAMs are highly expressed in tumor cells, and their density is correlated with various factors, which can be used as a new targeted therapy.^[29]^

According to our results, CD16 macrophages were expressed in gastric cancer and marginal tissues, whereas CD163 macrophages were highly expressed in gastric cancer and lowly expressed in marginal tissues, suggesting that the tumor-associated macrophages were closely correlated with gastric cancer. The relationships between the expression levels of two markers and clinicopathological factors (gender, age, tumor size, lesion site, degree of differentiation, depth of infiltration, lymph node metastasis, and TNM stage) were further analyzed. Besides, their correlations with the occurrence and development of gastric cancer were discussed. CD163 was suggested to be significantly correlated with gastric cancer differentiation, invasion depth, lymph node metastasis, and TNM stage (*P* < .05); while CD16 was not significantly correlated with each clinicopathological factor of gastric cancer (*P* > .05).

Based on Cox regression analyses on CD16- and CD163-related factors in gastric cancer, tumor size, differentiation, invasion depth, lymph node metastasis, TNM stage, CD16 and CD163 macrophage densities were the independent prognostic factors for gastric cancer patients, and the difference was statistically significant (*P* < .05). However, there was no significant correlation of gender, age or lesion location with the prognosis for patients with gastric cancer, and the difference was not statistically significant (*P* > .05). Staging of gastric cancer is the most important index for prognosis judgment, and lymph node metastasis always indicates poor prognosis. Meanwhile, the expression of CD163 macrophages is positively related to the distant lymph node metastasis.

Findings in this study also found that, CD163 expression in gastric cancer was significantly higher than that in marginal tissues, and the expression of HP-positive CD163 (75.38%) in gastric cancer was also higher than that in HP-negative tissues (52.00%). HP infection is identified to promote the proliferation of gastric mucosal epithelial cells and increase the risk of gastric cancer, which is consistent with literature reports.^[36]^ Moreover, HP infection induces the hyperplasia of gastric mucosal cells. Apart from its own virulence factors, HP infection may also be related to the following factors. The urease in HP breaks down urea in gastric juice to produce ammonia, while ammonia stimulates cell division and proliferation. HP also contains fatty acids, which damage cell membranes and cause cell death, thereby stimulating the renewal and acceleration of epithelial cells.^[37]^ HP infection renders inflammatory cells to produce cytokines, which stimulates cell proliferation. During the long process of inflammation, epithelial cells subjected to repeat damage and repair are prone to structural and functional changes as well as DNA damage, which thus produce a large number of active metabolites to induce mitosis and provide endogenous mutagens. In addition, the HP-infected gastric mucosa achieves pathological apoptosis through changes in apoptotic genes.^[38]^ The imbalance between the proliferation and apoptosis of epithelial cells may determine the final result after HP infection, namely, the reduced apoptosis and activated cell proliferation, which increases the risks of spontaneous DNA replication errors and tumors.^[39]^

In addition, this study also analyzed the correlations of CD16 and CD163 protein expression with HP infection, and confirmed the presence of a significant positive correlation between HP infection and CD163 protein expression (*r* = 4.556, *P* < .05). In addition, HP infection was obviously related to CD16 protein expression (*r* = 0.055, *P* > .05), suggesting that HP infection might promote the development of gastric cancer by affecting CD163 protein expression. HP disrupts the regulation of mitochondrial transcription genes and affects the transcriptional balance of oncogenes in gastric mucosal epithelial cells, but it makes no difference to the immune balance of T lymphocytes. HP is colonized on human gastric mucosal surface for a long time, and the body initiates an immune response to bacteria, resulting in immune or inflammatory tissue damage. Thus, the immune system in the body is activated through bacterial adhesion to the epithelium, thereby effectively presenting the HP antigen components to T cells and B cells, and generating the specific cellular and humoral immunity against HP.^[30]^ With regard to the HP whole bacteria, flagellum protein, lipopolysaccharide, urease, vacuolar toxin and toxin-related proteins can be used as immunogens, which can cause immune response in the body. The first stage of immune response is antigen presentation,^[40,41]^ and the host is usually completed by the antigen presenting cells. antigen presenting cells capture and process the antigens, then present them to the antigen-specific lymphocytes and the professional antigen-presenting cells, including dendritic cells (DCs), monocytes, macrophages, and B cells. Under normal circumstances, the immune system in the body has a perfect immune surveillance function. Among them, the antigen-presenting cells are dominated by DCs, which can recognize, capture, process, and present endogenous and exogenous antigens. In addition, DC also induce the body to produce specific anti-tumor and anti-inflammatory immune responses timelyr; at the same time, it has immunoregulatory functions.^[42]^ DCs are distributed in the gastric mucosa epithelium, lamina propria and corresponding lymphoid tissues. Besides, they are affected and regulated by local macrophages, cytokine and endotoxins, and are closely related to digestive tract inflammation and tumors. These results show that, the above-mentioned changes in immune function play an important role in the occurrence and development of gastric cancer, yet its underlying mechanism needs to be further studied.

## 5 Conclusions

Our current study highlights that the infection rate of HP in gastric cancer tissues is significantly higher, and the density of CD163 macrophages is not correlated with the general condition of tumor patients, but with tumor size, tumor differentiation, lymphatic metastasis, depth of invasion and TNM stage. More work is still needed regarding the regulation mechanism, yet our current study sheds more lights on the impact of HP on the phenotypic differentiation of TAMs, which can provide potential effective therapeutic approaches to improve prognosis, and it is of important clinical significance.

## Author contributions

**Conceptualization:** Ligao Wu.

**Data curation:** Qing Zhu, Xia Wu, Mingyang Tang.

**Formal analysis:** Qing Zhu.

**Funding acquisition:** Qing Zhu, Ligao Wu.

**Investigation:** Qing Zhu.

**Methodology:** Qing Zhu, Xia Wu, Mingyang Tang.

**Resources:** Qing Zhu, Xia Wu.

**Software:** Qing Zhu, Mingyang Tang.

**Validation:** Qing Zhu.

**Visualization:** Qing Zhu, Xia Wu.

**Writing – original draft:** Qing Zhu.

**Writing – review & editing:** Ligao Wu.

## References

[1] Sitarz R Skierucha M Mielko J Gastric cancer: epidemiology, prevention, classification, and treatment. Cancer Manag Res 2018;10:239. 29445300 10.2147/CMAR.S149619 PMC5808709

[2] Chen W Zheng R Baade PD Cancer statistics in China, 2015. CA Cancer J Clin 2016;66:115–32. 26808342 10.3322/caac.21338

[3] Sugano K Tack J Kuipers EJ Kyoto global consensus report on Helicobacter pylori gastritis. Gut 2015;64:1353–67. 26187502 10.1136/gutjnl-2015-309252 PMC4552923

[4] Malfertheiner P Megraud F O’morain C Management of Helicobacter pylori infection—the Maastricht V/Florence consensus report. Gut 2017;66:6–30. 27707777 10.1136/gutjnl-2016-312288

[5] Lu J Chen Y Liu Y Clinical significance of prognostic score based on age, tumor size, and grade in gastric cancer after gastrectomy. Cancer Manag Res 2018;10:4279. 30349362 10.2147/CMAR.S171663 PMC6183590

[6] Katai H Ishikawa T Akazawa K Five-year survival analysis of surgically resected gastric cancer cases in Japan: a retrospective analysis of more than 100,000 patients from the nationwide registry of the Japanese Gastric Cancer Association ( 2001– 2007). Gastric Cancer 2018;21:144–54. 28417260 10.1007/s10120-017-0716-7

[7] Deng J Yamashita H Seto Y Increasing the number of examined lymph nodes is a prerequisite for improvement in the accurate evaluation of overall survival of node-negative gastric cancer patients. Ann Surg Oncol 2017;24:745–53. 27770340 10.1245/s10434-016-5513-8

[8] Di Bartolomeo M Pietrantonio F Rulli E Impact on survival of timing and duration of adjuvant chemotherapy in radically resected gastric cancer. Tumori 2016;102:e15–9. 27032700 10.5301/tj.5000480

[9] Macalindong SS Kim KH Nam B-H Effect of total number of harvested lymph nodes on survival outcomes after curative resection for gastric adenocarcinoma: findings from an eastern high-volume gastric cancer center. BMC Cancer 2018;18:73. 29329569 10.1186/s12885-017-3872-6 PMC5766983

[10] Bilici A Selcukbiricik F Seker M Prognostic significance of metastatic lymph node ratio in patients with pN3 gastric cancer who underwent curative gastrectomy. Oncol Res Treat 2019;42:204–11. 10.1159/000496746 30870846

[11] Mirkin KA Hollenbeak CS Wong J Greater lymph node retrieval improves survival in node-negative resected gastric cancer in the United States. J Gastric Cancer 2017;17:306–18. 29302371 10.5230/jgc.2017.17.e35 PMC5746652

[12] Strong VE Wu Aw Selby LV Differences in gastric cancer survival between the US and China. J Surg Oncol 2015;112:31–7. 26175203 10.1002/jso.23940 PMC4667726

[13] Zhang K Shi H Xi H Genome-wide lncRNA microarray profiling identifies novel circulating lncRNAs for detection of gastric cancer. Theranostics 2017;7:213. 28042329 10.7150/thno.16044 PMC5196898

[14] Liu L Yi H He H Tumor associated macrophage-targeted microRNA delivery with dual-responsive polypeptide nanovectors for anti-cancer therapy. Biomaterials 2017;134:166–79. 28463694 10.1016/j.biomaterials.2017.04.043

[15] Kim J Bae J-S Tumor-associated macrophages and neutrophils in tumor microenvironment. Mediators Inflamm 2016;6058147. 26966341 10.1155/2016/6058147 PMC4757693

[16] Mantovani A Marchesi F Malesci A Tumour-associated macrophages as treatment targets in oncology. Nat Rev Clin Oncol 2017;14:399. 28117416 10.1038/nrclinonc.2016.217 PMC5480600

[17] Zheng H Li J Wang M Exhausting tumor associated macrophages with sialic acid-polyethyleneimine-cholesterol modified liposomal doxorubicin for enhancing sarcoma chemotherapy. Int J Pharm 2019;558:187–200. 30654062 10.1016/j.ijpharm.2019.01.005

[18] Zhou S Zhang T Peng B Targeted delivery of epirubicin to tumor-associated macrophages by sialic acid-cholesterol conjugate modified liposomes with improved antitumor activity. Int J Pharm 2017;523:203–16. 28336455 10.1016/j.ijpharm.2017.03.034

[19] Cheng Y Zhu Y Xu J PKN2 in colon cancer cells inhibits M2 phenotype polarization of tumor-associated macrophages via regulating DUSP6-Erk1/2 pathway. Mol Cancer 2018;17:13. 29368606 10.1186/s12943-017-0747-z PMC5784528

[20] Niu Z Shi Q Zhang W Caspase-1 cleaves PPAR( for potentiating the pro-tumor action of TAMs. Nat Commun 2017;8:766. 28974683 10.1038/s41467-017-00523-6 PMC5626701

[21] Binnemars-Postma K Bansal R Storm G Targeting the Stat6 pathway in tumor-associated macrophages reduces tumor growth and metastatic niche formation in breast cancer. FASEB J 2017;32:969–78. 10.1096/fj.201700629R 29066614

[22] Song Y Tang C Yin C Combination antitumor immunotherapy with VEGF and PIGF siRNA via systemic delivery of multi-functionalized nanoparticles to tumor-associated macrophages and breast cancer cells. Biomaterials 2018;185:117–32. 30241030 10.1016/j.biomaterials.2018.09.017

[23] Malik L Role of immunotherapy in bladder cancer: past, present and future. Cancer Chemother Pharmacol 2018;81:629–45. 29368051 10.1007/s00280-018-3518-7

[24] Mentis A-FA Boziki M Grigoriadis N Helicobacter pylori infection and gastric cancer biology: tempering a double-edged sword. Cell Mol Life Sci 2019;76:2477–86. 30783683 10.1007/s00018-019-03044-1 PMC11105440

[25] Elsebaey MA Tawfik MA Elshweikh SA Impact of helicobacter pylori infection on gastric variceal bleeding among patients with liver cirrhosis. Gastroenterol Res Pract 2019;2019 6529420. 10.1155/2019/6529420 PMC6387698 30881448

[26] Eda H Fukui H Uchiyama R Effect of Helicobacter pylori infection on the link between GLP-1 expression and motility of the gastrointestinal tract. PLoS One 2017;12:e0177232. 28545056 10.1371/journal.pone.0177232 PMC5436696

[27] Cover TL Helicobacter pylori diversity and gastric cancer risk. MBio 2016;7:e01869–915. 26814181 10.1128/mBio.01869-15 PMC4742704

[28] Lim JH Kim SG Choi JM Helicobacter pylori is associated with miR-133a expression through promoter methylation in gastric carcinogenesis. Gut Liver 2018;12:58. 28950691 10.5009/gnl17263 PMC5753685

[29] Gordon SR Maute RL Dulken BW PD-1 expression by tumour-associated macrophages inhibits phagocytosis and tumour immunity. Nature 2017;545:495. 28514441 10.1038/nature22396 PMC5931375

[30] Capitani N Codolo G Vallese F The lipoprotein HP 1454 of Helicobacter pylori regulates T-cell response by shaping T-cell receptor signalling. Cell Microbiol 2019;21:e13006. 30646431 10.1111/cmi.13006

[31] Biswas SK Mantovani A Macrophage plasticity and interaction with lymphocyte subsets: cancer as a paradigm. Nat Immunol 2010;11:889. 20856220 10.1038/ni.1937

[32] Zhu X Wang X Ma J Down-regulation of miR-1236-3p is correlated with clinical progression and unfavorable prognosis in gastric cancer. Eur Rev Med Pharmacol Sci 2018;22:5914–9. 30280772 10.26355/eurrev_201809_15920

[33] Shen F Liu P Xu Z CircRNA_001569 promotes cell proliferation through absorbing miR-145 in gastric cancer. J Biochem 2018;165:27–36. 10.1093/jb/mvy079 30304349

[34] Zhou L Yang Y Zhang R CircRNA_0023642 promotes migration and invasion of gastric cancer cells by regulating EMT. Eur Rev Med Pharmacol Sci 2018;22:2297–303. 29762831 10.26355/eurrev_201804_14818

[35] Ouyang Y Li Y Huang Y CircRNA circPDSS1 promotes the gastric cancer progression by sponging miR-186-5p and modulating NEK2. J Cell Physiol 2019;234:10458–69. 30417526 10.1002/jcp.27714

[36] Hatakeyama M Helicobacter pylori and gastric carcinogenesis. J Gastroenterol 2009;44:239–48. 19271114 10.1007/s00535-009-0014-1

[37] Jenkins D Charles I Thomsen LLe Roles of nitric oxide in tumor growth. Proc Natl Acad Sci U S A 1995;92:4392–6. 7538668 10.1073/pnas.92.10.4392 PMC41950

[38] Moss S Calam J Agarwal B Induction of gastric epithelial apoptosis by Helicobacter pylori. Gut 1996;38:498–501. 8707076 10.1136/gut.38.4.498 PMC1383103

[39] Correa P Miller MJ Helicobacter pylori and gastric atrophy-cancer paradoxes. J Natl Cancer Inst 1995;87:1731–2. 7473824 10.1093/jnci/87.23.1731

[40] Ermak TH Giannasca PJ Nichols R Immunization of mice with urease vaccine affords protection against Helicobacter pylori infection in the absence of antibodies and is mediated by MHC class II–restricted responses. J Exp Med 1998;188:2277–88. 9858514 10.1084/jem.188.12.2277 PMC2212427

[41] Pappo J Torrey D Castriotta L Helicobacter pylori infection in immunized mice lacking major histocompatibility complex class I and class II functions. Infect Immun 1999;67:337–41. 9864234 10.1128/iai.67.1.337-341.1999 PMC96315

[42] Hamblin T From dendritic cells to tumour vaccines. Lancet 1996;347:705–6. 10.1016/s0140-6736(96)90071-9 8601996

